# A novel CCBE1 mutation leading to a mild form of hennekam syndrome: case report and review of the literature

**DOI:** 10.1186/s12881-015-0175-0

**Published:** 2015-04-30

**Authors:** Patrick Frosk, Bernard Chodirker, Louise Simard, Wael El-Matary, Ana Hanlon-Dearman, Jeremy Schwartzentruber, Jacek Majewski, FORGE Canada Consortium, Cheryl Rockman-Greenberg

**Affiliations:** Department of Pediatrics and Child Health, University of Manitoba, FE229 Community Services Bldg, 685 William Ave, Winnipeg, MB R3E 0Z2 Canada; Department of Biochemistry and Medical Genetics, University of Manitoba, Winnipeg, MB Canada; McGill University and Genome Quebec Innovation Centre, QC, Canada

**Keywords:** CCBE1, Hypoalbuminemia, Lymphangiectasia, Lymphatic Dysplasia, Polydactyly, Protein-losing Enteropathy

## Abstract

**Background:**

Mutations in *CCBE1* have been found to be responsible for a subset of families with autosomal recessive Hennekam syndrome. Hennekam syndrome is defined as the combination of generalized lymphatic dysplasia (ie. lymphedema and lymphangiectasia), variable intellectual disability and characteristic dysmorphic features. The patient we describe here has a lymphatic dysplasia without intellectual disability or dysmorphism caused by mutation in *CCBE1*, highlighting the phenotypic variability that can be seen with abnormalities in this gene.

**Case presentation:**

Our patient is a 5 week old child of Pakistani descent who presented to our center with generalized edema, ascites, and hypoalbuminemia. She was diagnosed with a protein losing enteropathy secondary to segmental primary intestinal lymphangiectasia. As the generalized edema resolved, it became clear that she had mild persistent lymphedema in her hands and feet. No other abnormalities were noted on examination and development was unremarkable at 27 months of age. Given the suspected genetic etiology and the consanguinity in the family, we used a combination of SNP genotyping and exome sequencing to identify the underlying cause of her disease. We identified several large stretches of homozygosity in the patient that allowed us to sort the variants found in the patient’s exome to identify p.C98W in *CCBE1* as the likely pathogenic variant.

**Conclusions:**

*CCBE1* mutation analysis should be considered in all patients with unexplained lymphatic dysplasia even without the other features of classic Hennekam syndrome.

## Background

Hennekam syndrome (OMIM#235510) is a disorder of lymphatic development that includes lymphedema, lymphangiectasia, characteristic dysmorphism and cognitive impairment [1]. The dysmorphic features include a flat nasal bridge, hypertelorism, and small mouth. Approximately 25% of patients who have Hennekam syndrome were found to have autosomal recessive mutations in *CCBE1* [2] and a further 20% of patients have mutations in *FAT4* [3]. *CCBE1* is clearly important for lymphatic development based on animal models [4,5] however the connection of *FAT4* to lymphatic development is still obscure.

Our understanding of the phenotype associated with *CCBE1* mutation has evolved over time. The original report noted that patients with Hennekam syndrome demonstrate significant variability in the degree of cognitive impairment (ranging from normal to moderate impairment) [2]. However, all of the original reported patients had the severe lymphatic abnormalities and characteristic dysmorphic features. A recent report compared individuals with a clinical diagnosis of Hennekam Syndrome with and without mutations in *CCBE1* [6]. This article described another three patients with no developmental difficulties although all had intestinal lymphangiectasia, appendicular lymphedema and dysmorphic features. Other reports of patients with *CCBE1* mutations have classified a few families as having “generalized lymphatic dysplasia”. One family included three affected individuals, all of whom had hydrops fetalis, and two of whom had died in early life [7]. The proband of the latter family, at the age of 6 years, had normal development but most of the other features were consistent with Hennekam syndrome (massive lymphedema, gut lymphangiectasia, and dysmorphic features consistent with *in utero* edema). The second family had an affected sib pair with normal development, gut lymphangiectasia, subtle dysmorphisms, and moderate lymphedema [8]. There is one report that suggests *CCBE1* may also be a cause of lymphedema-cholestasis syndrome (OMIM#214900), although it isn’t clear if this was the coincidental finding of Hennekam along with cholestasis or whether *CCBE1* mutation was the underlying cause of the cholestasis as well, perhaps secondary to a lymphatic effect [9].

Given that most of the consistent features of Hennekam syndrome are felt to be secondary to disordered lymphatic development *in utero* [1], it seems reasonable to conclude that these patients all represent the same underlying pathogenetic mechanism, just with varying severity. The patient we present here is likely the mildest to be reported to date with mutations in *CCBE1* which suggests Hennekam syndrome has an even broader phenotypic variability than previously thought.

## Case report

This female infant was born to a 29 year old G2P1 mother at term via an emergency C-section due to fetal distress. During the prenatal period, an echogenic intracardiac focus and nuchal thickening (6-7 mm) were identified at 21 weeks gestation. The family received genetic counseling and further investigations were declined. Apgar scores were 8 at 1 minute and 8 at 5 minutes and the infant fed well shortly after birth. Postnatal exam showed a healthy appearing infant with bilateral type B postaxial polydactyly of the hands (postminimus). She did well in this immediate post-natal period and, after removal of the polydactyly, was discharged from the hospital. At home she was fed breast milk but, even with adequate volumes grew poorly. She was admitted to our hospital at 26 days of age with severe failure to thrive and a history of diarrhea and occasional emesis. Serum albumin levels during this admission ranged from 13 – 17 g/L (normal range 32 – 48 g/L). When she was switched to a hypo-allergenic formula, she started gaining weight and was discharged with a provisional diagnosis of cow’s milk protein allergy. She presented again at 51 days of age with significant ascites, generalized edema and severe hypoalbuminemia with a serum albumin level of 10 g/L. The remainder of her examination was unremarkable. Large volume, non-bloody diarrhea had continued to be a problem throughout the time at home despite the change in formula.

Her investigations included a normal CBC with normal lymphocytic count, normal liver enzymes, normal bilirubin, low serum transferrin, low serum immunoglobulins and normal thyroglobulin. Her stools were negative for fat globules and reducing substances. However, 24 hour-stool collection had a markedly elevated alpha-1-antitrypsin level of 576 mg/g (normal <54 mg/g) indicative of protein losing enteropathy. Esophagogastroduodenoscopy and sigmoidoscopy were performed and did not reveal any obvious abnormalities. Biopsies taken at the same time were also unremarkable by both light and electron microscopy. A small bowel contrast study showed thickening and nodularity in several segments of the small bowel suggestive of segmental intestinal lymphangiectasia. Treatment during her admission included multiple albumin infusions and eventually a switch to a medium chain triglyceride (MCT) containing formula. She responded very well with resolution of vomiting and diarrhea. At discharge, the ascites had resolved as had most of the edema and she was gaining weight appropriately. At her last clinical assessment she was 27 months of age and her albumin levels were maintained at 25-30 g/L on a MCT rich-diet. With the resolution of the facial edema evident in hospital she was non-dysmorphic based on the assessments of two experienced dysmorphologists (B.C. and C.R.G) and there was absence of distichiasis. Her height, weight, and occipitofrontal circumference were all between the 50th and 75th percentiles. Her hands and feet continued to be mildly edematous despite control of her albumin levels. Her development was considered normal with age appropriate verbal and motor skills and no social concerns or abnormal behaviors. Her development was assessed on the Bayley scales of infant and toddler development, 3rd edition (Bayley III) and showed a Cognitive Composite at the 25th centile, a Language Composite at the 34th centile and a Motor Composite at the 50th centile.

Her family history was significant for consanguinity with her parents being first cousins of Pakistani descent. There was an older healthy male sibling. A maternal aunt had an undiagnosed disorder which included corneal clouding and she died at 13 years of age. Genetic investigations in our patient were initiated early on in the course of her hospitalization and included a normal transferrin isoelectric focusing, normal plasma amino acids, normal urine organic acids and normal urine mucopolysaccharides. Her karyotype was 46,XX and an array CGH was done which identified an ~100 kb deletion on chromosome 15q11.2 that encompasses exon u1b of SNRPN (within the Prader-Willi / Angelman critical region). This was felt to be an incidental finding as it was inherited from her normal father and there was no overlap of her symptoms with classic Prader-Willi syndrome.

**Fig. 1:**
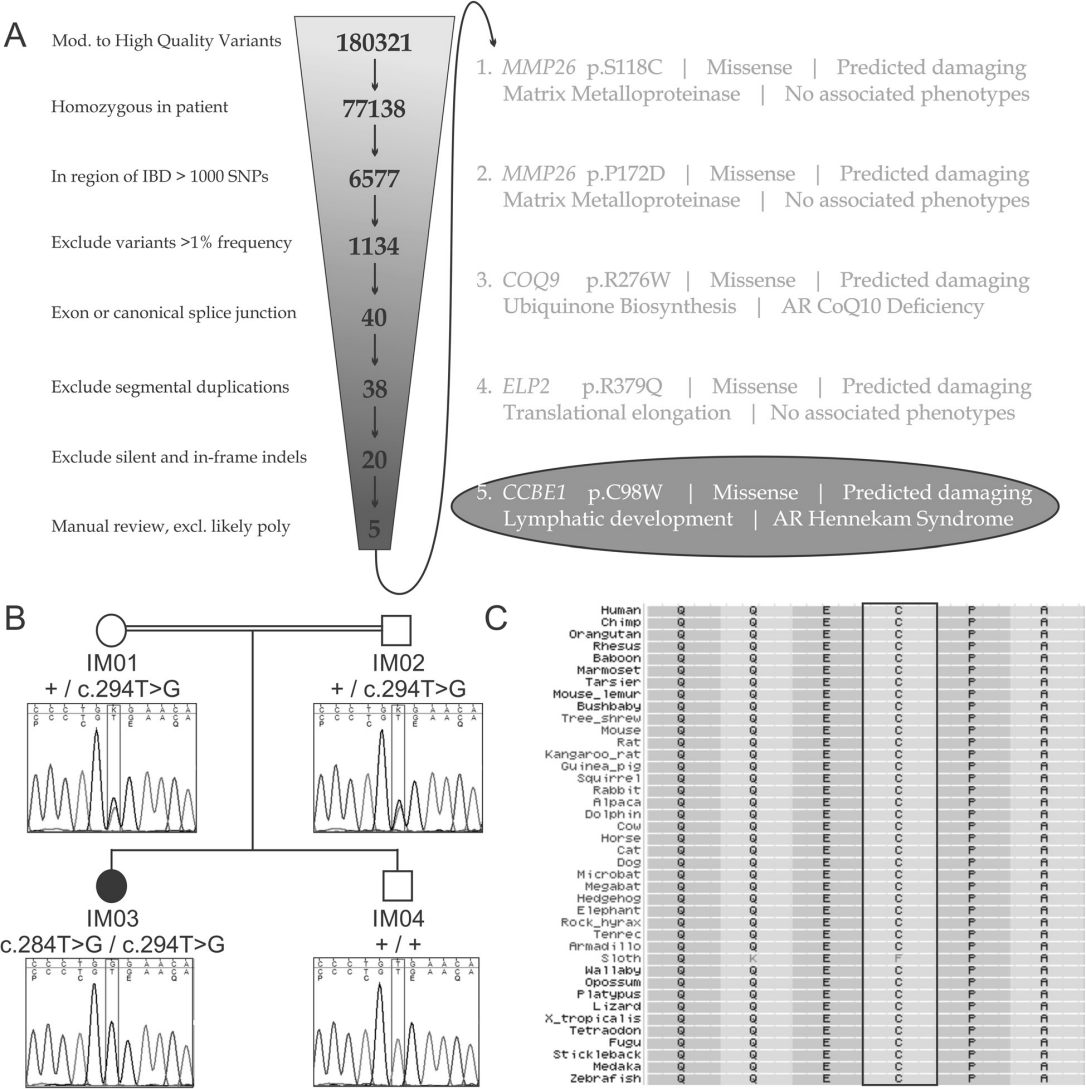
Molecular analysis identifying a mutation in *CCBE1* as the likely cause of disease. **(a)** Exome sequencing data from patient IM03. Filter criteria are as shown. The candidates eliminated on manual review were largely known polymorphisms that were not called appropriately by the annotation algorithm. Five candidates could not be definitively excluded. The most likely candidate for the disease-causing mutation is in CCBE1 given the known function of the gene and its association with a related phenotype in humans (Hennekam Syndrome). **(b)** Confirmatory Sanger sequencing of the CCBE1 variant identified through the exome analysis. The variant is a T > G transversion at position 294 of the coding sequence (c.294 T > G). This changes codon 98 from a cysteine to a tryptophan (p.C98W). In the above figure, + represents wild type sequence. On the electropherograms the upper portion represents the actual sequence read and the lower portion represents the expected sequence along with the translation. **(c)** Multiple protein alignment with CCBE1 and its orthologs (reverse orientation). The affected amino acid is within the rectangle. This cysteine is highly conserved amongst different organisms and the mutation is predicted to be deleterious to CCBE1 function

An autosomal recessive lymphatic dysplasia was strongly suspected given the consanguinity. In order to identify the causative mutation, we used a combination of SNP genotyping and exome sequencing. The nuclear family was genotyped using Illumina Human Omni 2.5 SNP arrays at the Centre for Applied Genomics, Toronto, Canada. Twenty-seven regions of homozygosity containing >1000 SNPs were evident in the patient sample and ranged in size from 0.9-37.5 Mb. Segregation analysis was used to rule out some of these regions, leaving ~183 Mb of sequence that appeared to be identical by descent (IBD) but not present in the unaffected brother. Exome sequencing was performed on the affected patient as described for previous FORGE projects [10]. Mean Consensus Coding Sequence (CCDS) coverage was 132X with 93% of bases covered by at least 20 reads. Approximately 180,000 moderate to high quality variants were identified in the sample. Using the regions of IBD and other filters consistent with our genetic model, we were able to narrow the candidates down to 5 possibilities (Fig. 1a). The most likely candidate, based on previous functional data, was a homozygous missense variant in CCBE1 (c.294 T > G, p.C98W [NM_133459]). This variant segregates in the family with both parents being carriers (Fig. 1b). This variant is rare (not found in dbSNP or the NHBLI exome server) and affects a highly conserved amino acid found within the EGF calcium-binding domain. Orthologs from 38/39 species, ranging from placental mammals to fish, show conservation of this residue suggesting functional importance (Fig. 1c).

Collagen- and calcium-binding domain 1 (CCBE1) is a signaling molecule essential for lymphangiogenesis in humans and model organisms. Initial description of CCBE1 function came from a zebrafish mutant, full of fluid (fof), identified on a forward genetic screen [4]. These fish had an absence of truncal lymphatic vessels, including the thoracic duct, and developed significant edema. The cause for this zebrafish mutant was determined to be a missense mutation within the CCBE1 EGF domain (p.D162E) suggesting this protein was essential for appropriate lymphangiogenesis. Creation of a knockout mouse allowed further elucidation of the function of CCBE1 [5]. CCBE1 knockout mice died prenatally with progressive edema beginning at E13.5 onward and lacked all detectable lymphatic vessels. The protein was found to be excreted and bound to the extracellular matrix, acting as a potentiating factor of VEGF-C in lymphatic budding and outgrowth.

Mutations in the *CCBE1* gene have been found in patients with Hennekam syndrome of varying severity. Our patient appears to represent the mildest of this phenotypic spectrum. Certainly, the mutation identified in her (p.C98W) is similar to missense mutations that have been identified in other Hennekam patients (eg. p.C47F, p.C75S, p.C102S, p.R158C, p.C174R, and p.C174Y) [2, 6]. All of these mutations affect cysteine residues within the EGF domain and given the disulfide linkages within EGF domains, it is reasonable to assume all of them will disrupt the tertiary structure of the domain to a certain degree. Three of these previously reported mutations (p.C75S, p.C102S, and p.C174R) were studied further and had reduced ability to rescue zebrafish ccbe1 knockdown morphants, confirming their pathogenicity [2]. Further functional work is needed to determine if the p.C98W allele is the reason for her milder phenotype or whether there are alternate genetic or environmental factors at play.

The outstanding issue in this case is the preaxial polydactyly of her hands. No other individuals in the family have polydactyly (either currently or treated at birth like our patient). This anomaly has never been seen in *CCBE1*-related disease nor has it been associated with the clinical entity of Hennekam syndrome to our knowledge. Postaxial polydactyly has been reported to be associated with intestinal lymphangiectasia in Urioste syndrome, however, this clinical entity also includes hepatic failure, persistence of the Mullerian ducts in males and all reported patients have died within 1 year of age [11–13]. The underlying cause of Urioste syndrome remains unknown but it does seem unlikely to be associated with mutation in *CCBE1* given the lack of any Mullerian abnormalities or hepatic failure in the known *CCBE1* patients [6]. It is possible that the polydactyly in our patient is unrelated to the lymphatic disorder. We did review her molecular data for other variants that could independently cause polydactyly via a candidate gene approach but we were unsuccessful. At this point it is unclear whether this feature is related to her *CCBE1* mutation or not.

## Conclusions

Hennekam syndrome is clearly a disorder of lymphatic development. The classic phenotype includes lymphangiectasia (particularly of the gut), lymphedema, characteristic dysmorphic features, and intellectual disabilities. Certainly the lymphangiectasia and lymphedema are directly related to lymphatic development. It seems likely the dysmorphic features (round flat face, flat bridge of the nose, hypertelorism, and small mouth) are all secondary to *in utero* lymphatic perturbations when the facial structures are being formed. We cannot exclude the possibility that there is some direct effect on facial morphogenesis from the CCBE1 mutation itself, however, the lack of dysmorphisms in our patient argues against this. It is possible that even the cognitive impairments are secondary to *in utero* lymphatic abnormalities as this is the least consistent feature of the syndrome and is usually only present in the most severe cases. The abnormal lymphatics could have a secondary effect on neuronal development if they were severe enough during the appropriate stage in gestation.

Our patient is likely the mildest to be reported with mutations in *CCBE1* to date. She has segmental gut lymphangiectasia that is well controlled on a MCT diet. Her development is unaffected, she has no dysmorphic features and there is minimal acral lymphedema. She had some *in utero* involvement given the nuchal thickening of 6-7 mm noted prenatally but it clearly did not have a large phenotypic consequence. This report extends the phenotype of Hennekam syndrome to include even milder lymphatic dysplasias. We suggest that the term Hennekam syndrome be used for a spectrum of autosomal recessive lymphatic dysplasias that encompass the “classic” form with more severe *in utero* effects (hydrops fetalis, dysmorphism, cognitive impairment, gut and other organ lymphangiectasia, severe lymphedema) to a milder form as presented here (gut lymphangiectasia, mild lymphedema). The presence of both lymphangiectasias (particularly of the gut) and lymphedema would differentiate Hennekam syndrome from other congenital primary lymphatic dysplasias like Milroy syndrome (isolated acral lymphedema) and isolated intestinal lymphangiectasia [14]. Our case highlights the need for a very detailed clinical assessment in patients with any suggestion of a lymphatic abnormality. Testing should be directed by phenotype but *CCBE1* sequencing should be considered in even apparently isolated cases of intestinal lymphangiectasia as the presence of lymphatic abnormalities outside of the gut may be subtle.

## Consent

Written informed consent was obtained from the patient’s parents for publication of this Case report and any accompanying images. A copy of the written consent is available for review by the Editor of this journal.

## Abbreviations

CCDS: Mean consensus coding sequence
IBD: Identity by descent
MCT: Medium chain triglyceride
